# Melatonin Use in Pediatrics: A Clinical Review on Indications, Multisystem Effects, and Toxicity

**DOI:** 10.3390/children11030323

**Published:** 2024-03-09

**Authors:** Pranita Shenoy, Adriana Etcheverry, Jalyn Ia, Manisha Witmans, Mary Anne Tablizo

**Affiliations:** 1 Department of Pediatrics, Valley Children’s Hospital, Madera, CA 93636, USA; pshenoy1@valleychildrens.org (P.S.); aetcheverry1@valleychildrens.org (A.E.); mtablizomd@stanford.edu (M.A.T.); 2 Department of Ecology and Evolutionary Biology, University of California, Los Angeles, Los Angeles, CA 90095, USA; 3 Department of Pediatrics, University of Alberta, Edmonton, AB T6G 2B7, Canada; 4 Department of Pediatrics, Stanford University, Palo Alto, CA 94304, USA

**Keywords:** melatonin, pediatric indications, adverse effects and poisoning

## Abstract

Exogenous melatonin is typically used for sleep regulation in the context of insomnia either in healthy children or those with neurodevelopmental disabilities. It is also used for the management of circadian rhythm sleep disorders in pediatric and adolescent patients. There are also many other possible indications that we will discuss in this paper beyond the role of melatonin for sleep regulation, including its potential use for various areas of medicine such as inflammatory conditions. Since melatonin is unregulated in the United States, distributed over the counter and perceived to be natural and safe, it has become available in many forms in the last two decades. With increasing sleep disturbances and mental health problems after the COVID-19 pandemic, melatonin has become even more popular and studies have shown a dramatic increase in use as well as resulting side effects, including melatonin overdose. As melatonin is generally viewed by physicians as a benign medication, we hope to increase awareness of melatonin’s properties as well as negative side effects to optimize its use in the pediatric population.

## 1. Introduction

Melatonin is a naturally occurring hormone synthesized by the pineal gland. The synthetic manufactured forms have prevalent use in pediatric medicine as a medication to treat sleep disorders because of its perceived safety profile and minimal side effects. In fact, synthetic melatonin preparations are widely available over the counter (OTC) in the United States with sales increasing from $285 million in 2016 to $821 million in 2020 [1]. 

In the wake of increasing sleep disturbances and mental health conditions after the COVID-19 pandemic, melatonin’s popularity has increased dramatically despite the lack of rigorous evidence. 

Melatonin’s primary role is in sleep and circadian rhythm regulation. The literature on melatonin use in the pediatric population generally focuses on its role on delayed sleep phase syndrome (DSPS) and sleep onset insomnia especially in neurodevelopmental disorders such as autism spectrum disorder (ASD), attention-deficit/hyperactivity disorder (ADHD), and epilepsy [2]. It also has been reported to have protective mechanisms against oxidative stress and has been shown to affect inflammatory response and immune cytokines [3]. Its use has a number of advantages and disadvantages which warrant further exploration. This paper will address some of the important issues related to melatonin indications, varied dosing and formulations, physiological effects of its use, adverse events, and poison control reporting in pediatric medicine.

### 1.1. Melatonin Pharmacology in Children

Melatonin (5-methoxy-N-acetyltryptamine) is an endogenous hormone secreted from the pineal gland synthesized via N-acetylation and O-methylation of serotonin. Synthesis and secretion of endogenous melatonin is controlled by the hypothalamus which is activated by external factors like darkness [4]. Efferent nerves from the suprachiasmatic nucleus (SCN) project to the paraventricular nucleus in the hypothalamus which then project axons to the spinal cord in the upper thoracic region and superior cervical ganglion, subsequently innervating the pineal gland [5]. 

In step with circadian rhythm, melatonin levels in the pineal gland generally rise at sleep onset and fall at sleep offset. In preparation for sleep, levels are highest, then decrease as the night progresses. Melatonin uses two G protein-coupled receptors (GPCRs) MT1 and MT2 which are located in many areas of the human brain including SCN, cerebellum, thalamus, hippocampus and cortex [6]. Binding of melatonin to MT1 receptors promotes sleep onset by inhibiting SCN firing; binding of MT2 receptors shifts timing of the circadian system [4]. MT1 receptors are found mainly in the hypothalamus and cerebellum. However, MT1 can also be found peripherally in the aorta, spleen, placenta, and pancreas for example. MT2 is less widespread compared to MT1 receptors, mainly found in the SCN and retina but can also be found in immune system and reproductive tissues. These receptors are regulated differently based on physiological vs supraphysiological melatonin concentrations. Introducing exogenous sources of melatonin will increase the blood melatonin concentration, altering receptor sensitivity to melatonin. In the SCN in vitro, the density of receptors increases and the affinity of melatonin tends to decrease, although this was not seen in mammalian cells [7].

The pineal gland is the main source of melatonin but other organs such as the retina, platelets, and GI tract can also synthesize and secrete this hormone [8]. Pineal gland production of melatonin can be acutely and completely suppressed by light exposure as little as room light. Higher lux is associated with more suppression of melatonin in a dose response fashion. Selective Beta 1-adrenergic antagonist (atenolol, metoprolol), ethanol, and nonsteroidal anti-inflammatory medications can also reduce levels of plasma melatonin [5].

Light is detected by retinal photoreceptors and transmitted through the pineal gland where melatonin is secreted into the blood. Regulation is mainly via alpha 1 and beta 1 adrenoreceptors which affects activity of aralkylamine N-acetyltransferase (AANAT), tryptophan hydroxylase (TPH), and hydroxyindole-O-methyltransferase (HIOMT) via cyclic AMT. While the effects of the adrenoreceptors are to increase melatonin secretion, the MT receptors demonstrate negative feedback regulation on melatonin synthesis. One particular study showed that when activating the adrenergic receptors and inhibiting the MT receptors, there was a more exaggerated increase in melatonin secretion [8].

Looking at melatonin in the pediatric population, babies start to produce endogenous melatonin from their pineal gland around 3 months of age. If a baby is breastfed, however, they may have increased levels from breastmilk. Studies show that for each 1 mg of exogenous melatonin a mother took, the average increase in melatonin concentration in breastmilk would be 0.4 to 1 mcg/L [9]. Neonates born before 34 weeks gestation have lower concentrations of plasma melatonin than neonates born after 34 weeks. 

**Figure 1:**
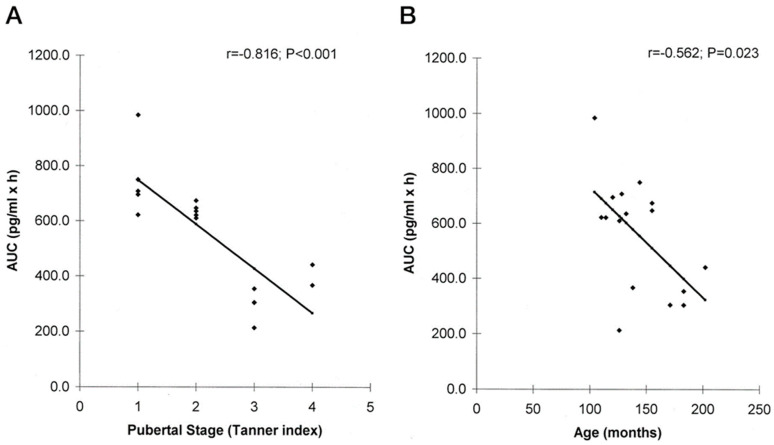
Stronger association of 12-h AUC of circulating melatonin as a function of pubertal stage (**A**) than as a function of chronological age (**B**) [10], Copyright © 2000 by The Endocrine Society.

In childhood, melatonin levels generally increase until puberty at sleep onset and then start to decline with age [10]. As seen in Figure 1, circulating melatonin differs in concentration in relation to pubertal stage and chronological age, with highest concentrations at a younger age [10]. Typically, children will have higher levels of melatonin production than adults, which is important to consider when considering supplementation [11].

### 1.2. Common Use of Melatonin in Sleep Disorders in Children

Exogenous melatonin, with its chronobiotic and hypnotic properties, has emerged as a pivotal intervention for the management of circadian rhythm sleep disorders in pediatric and adolescent patients. While not officially approved by the FDA, melatonin has garnered widespread use among pediatricians, psychiatrists, and sleep physicians, particularly for its efficacy in managing various sleep disorders. Its ability to ameliorate sleep-wake rhythm disturbances and reduce sleep latency makes it a valuable tool in addressing sleep-related issues in children, and adolescents [12]. Its primary applications include sleep onset insomnia and delayed sleep phase syndrome (DSPS) within the pediatric population, especially in neurodevelopmental disorders such as autism spectrum disorder (ASD) and attention-deficit/hyperactivity disorder (ADHD) [13].

Melatonin supplementation has demonstrated efficacy in advancing sleep onset time (SOT) and dim light melatonin onset (DLMO) and decreasing sleep onset latency (SOL) in pediatric patients with sleep onset insomnia. A meta-analysis from 2020 found that melatonin treatment significantly advanced SOT by 0.62 h compared to a placebo. Additionally, compared to the placebo group, melatonin treatment led to a significant advancement in DLMO by −0.82 h and SOL by −0.36 h [14]. Similarly, in delayed sleep phase syndrome (DSPS), characterized by deviations of 3–6 h in sleep onset and offset times from conventional sleep-wake timing, a review of exogenous melatonin administration in both adolescents and adults revealed an average advancement of endogenous melatonin secretion by 1.18 h, coupled with a notable reduction in average sleep latency by 23 min and a decrease in SOL by 23.27 min, reflecting enhanced sleep initiation in adolescents [15]. In a double blind randomized controlled trial, participants with clinically diagnosed delayed sleep-wake phase disorder who took 0.5 mg melatonin an hour prior to their desired sleep time with sleep and wake scheduling reported both subjective and objective improvement in sleep disturbances [16].

Melatonin supplementation holds promise in addressing sleep problems associated with neurodevelopmental disorders. Autism spectrum disorder (ASD), characterized by communication and social interaction challenges, frequently accompanies sleep disorders in 50% to 75% of cases. Research has shown that after 13 weeks of double-blind treatment, participants experienced an average increase in nighttime sleep duration. Those who received PedPRM, a form of melatonin treatment, increased an average of 57.5 min of additional sleep, while those who received a placebo experienced only a minor increase of 9.14 min in their sleep duration [17]. 

The evidence regarding the impact of melatonin on Attention-Deficit/Hyperactivity Disorder (ADHD) is somewhat mixed. While some studies do not demonstrate a large difference between the melatonin-treated group and the placebo group, multiple investigations suggest the potential for melatonin use in advancing sleep onset in children with ADHD. For instance, in one study, the melatonin-treated group experienced a modest advancement in sleep onset (averaging around 27 min earlier) compared to a delay in the placebo group (approximately 10.5 min). Additionally, the melatonin group showed a total increase in sleep duration, albeit not dramatically greater than the placebo group’s increase. It has also been found that in children with ADHD, there is often a delayed onset of melatonin peak by 2 h, so melatonin may also help shift sleep onset timing [13]. Although the evidence for significant improvements in ADHD-related sleep problems may not be as robust, the cumulative findings across various studies hint that melatonin offer benefit.

Because melatonin is used to improve sleep start time and lengthen sleep duration, it may have a role in treating parasomnias in children such as night terrors and sleepwalking. The effect of melatonin is not for terminating the parasomnia itself but rather better and longer sleep duration decreases the predisposition for parasomnias by improving sleep parameters. However, melatonin has also been linked to worsening of parasomnias in certain populations based on anecdotal evidence.

In adults, melatonin is also reported for use in various contexts by improving sleep continuity. Melatonin is considered a potential treatment for parasomnias, such as non-rapid eye movement (NREM) parasomnias. Melatonin may work in this case because sleep fragmentation or sleep deprivation are triggers for parasomnias and melatonin may mitigate this. With its good safety profile, melatonin can be an effective option for managing these sleep disorders, offering promising results compared to medications like z-drugs or antidepressants [18]. In adult patients with traumatic brain injury, a randomized controlled trial found that 2 mg of melatonin improved subjective sleep quality and reduced anxiety over a 4-week period [19]. Most recently, the American Academy of Sleep Medicine Guidelines for REM behavior disorder (RBD) have recommended the use of immediate release melatonin as second line treatment for isolated RBD in adults as well as for secondary RBD [20]. Studies on these topics have not been particularly researched in pediatrics. 

### 1.3. Other Potential Indications

Beyond its conventional roles in sleep regulation, melatonin’s influence in pediatrics encompasses broader physiological functions, shedding light on its potential benefits in specific situations. It is important to emphasize that while synthetic melatonin is categorized as a dietary supplement and lacks FDA approval for specific uses, FDA-approved melatonin receptor agonists like ramelteon and tasimelteon have been developed for insomnia [21].

Studies show promising results for melatonin in migraine management but there are limited data on its use in children and adolescents. In one study, the melatonin group had fewer migraine days than the placebo group in the final four weeks of treatment [22]. Another study also supports the effectiveness and tolerability of melatonin (3 mg) as a preventive therapy for migraines [23].

In the context of atopic dermatitis, emerging research has explored the potential benefits of melatonin supplementation in children and adolescents, shedding light on its role in reducing disease severity and improving sleep quality. In two small randomized trials involving children and adolescents with atopic dermatitis, melatonin supplementation showed positive outcomes. In the first trial, 48 children with atopic dermatitis experienced a greater reduction in the severity of their condition and a shorter time to fall asleep when taking melatonin (3 mg) compared to a placebo. No side effects were reported [24]. The second trial, which included 70 children aged 6 to 12 years, found that those taking melatonin (6 mg) had greater improvements in their skin condition and sleep quality compared to those on a placebo. Additionally, the melatonin group had lower levels of serum total IgE levels, a marker associated with atopy and allergies, and longer sleep duration, with no reported adverse effects. Melatonin appears to exert its effects on atopic dermatitis by potentially influencing B cells responsible for IgE antibody production, potentially reducing their activity. It may impact the release of specific cytokines, which are crucial for regulating and enhancing T helper immune responses, thereby potentially altering pathways associated with IgE production [25]. These results suggest that melatonin supplementation may be beneficial for managing atopic dermatitis in children and improving their sleep.

Melatonin also induces glial cell-line derived neurotrophic factor (GDNF) expression in neural stem cells, suggesting that melatonin is involved in early nervous system development. Studies in nonhuman primates show that maternal melatonin can stimulate the growth of the primate fetal adrenal gland and entraining fetal circadian rhythm, including SCN rhythms [26].

Melatonin can also serve as a protective mechanism against oxidation and cell death in human gametes. It may even improve the quality of embryos, which can potentially be useful in fertility practice, however, the literature is limited. Melatonin levels in utero rise with higher gestational age and with multiple fetuses. This rise seems to be related to placental melatonin levels. Studies have also shown that the reduction in melatonin exposure, such as for mothers who do night shift work, can disrupt behavior and physiology. It can therefore increase the risk of miscarriages, preterm delivery, and low birth weight [26]. The mechanism of this is unclear. However, failures in conception is postulated to be related to endometrial thickening, defects in phases of the menstrual cycle, and various disorders of the female reproductive tract. The cyclical nature of melatonin production contributes to improving implantation. In the blastocyst, it can indirectly stimulate corpus luteum activity and can alter endometrial thickening. Therefore indicating that melatonin has a potential to decrease the risk of failed conception and miscarriages. However, it is important to note that these studies are mainly completed with animal models, so translation to humans may differ [27].

The data suggests that the role of melatonin in circadian rhythms are important for neurodevelopment, including mood, behavior, development, and intellect. Furthermore, melatonin can be involved in blood pressure, autonomic cardiovascular regulation, immune system regulation, and various other physiological functions including free radical detoxification through MT3 receptors protecting the brain from oxidative stress. These antioxidant properties also protect the GI tract from ulcers by reducing the secretion of hydrochloric acid and oxidative effects of bile acids on the epithelium, as well as by increased mucosal secretions of bicarbonate via MT2 receptors [28].

Melatonin can increase bone mass by stimulating bone cell proliferation and type I collagen synthesis while inhibiting bone resorption. Additionally, it has been shown to have effects on reproduction and sexual maturation by downregulating GnRH gene expression in a cyclic pattern. The pulsatile nature of LH secretions can mediate seasonal changes of reproduction seen in animals, such as in seasonal breeding. This is only moderately observed in humans [28].

In the perioperative context, melatonin has been assessed as a treatment for preoperative and postoperative anxiety. A Cochrane review of 27 randomized controlled trials involving 2319 adults concluded that melatonin may have a similar effect to benzodiazepines in reducing preoperative anxiety [29].There are no studies yet for this indication in the pediatric population.

## 2. Dosing

Before considering melatonin supplementation, physicians should always discuss proper sleep hygiene such as maintaining consistent sleep schedule and limiting use of electronic devices before bedtime. Older children and teens should avoid taking naps and caffeine. Ensure that the bedroom is conducive for sleeping by making it comfortable, quiet, and dark with the appropriate room temperature. Exposure to light at night suppresses the onset of the body’s natural melatonin rise and can phase shift circadian timing. Therefore, light avoidance is a fundamental consideration in sleep hygiene. Other behavioral interventions include restricting bedtime to time of sleep onset and waking. Sleep diaries or actigraphy are used to determine best times for sleep onset and offset considering both behavioral and circadian factors. Many sleep onset behaviors can be improved by targeted sleep hygiene practices alone. Melatonin can act as a supplemental aid and its benefit should be assessed in the patient’s optimal sleep environment, ideally with some support of trained clinicians [30].

Melatonin is commercially available in an oral tablet, oral liquid, soft chew gummy, capsules, tea, lozenges, and oral spray in the United States. It crosses the blood-brain barrier and placenta. It is metabolized by the liver via oxidative metabolism using CYP1A, with minor roles of CYP2C19 and possibly CYP2C9, and is eliminated via renal excretion. For a non-smoking, non-geriatric patient, without hepatic or renal impairment, the mean half-life after oral administration for immediate-release is roughly 45 min. The time to reach maximum concentration after oral administration without food is 50 min, ranging from 15 to 210 min. With IV administration, the half-life is approximately 28 min [31]. The excretion of primary metabolite is complete within 12 h following a single dose of an extended-release product [32]. 

Administration of 0.3 mg of melatonin can reach levels similar to physiologic concentration, while doses above 1 mg are above physiologic levels. The higher doses (>10 mg) of oral melatonin can produce concentrations that can persist for more than 24 h with concentrations more than 100-fold higher than those normally found in adults [5]. 

The optimal dosing of exogenous melatonin in children requires careful consideration, as it should be tailored to each child’s specific needs, age, and the indication being treated. While universal dosing and administration timing guidelines for melatonin in healthy pediatric patients remain unestablished, according to the findings in a recent review, the proposed dosage of melatonin to healthy children is 0.5 to 5 mg, approximately 30 min to 1 h before bedtime [14,33]. 

In the United States, melatonin products formulated for pediatric use suggest dosing from 0.5 to 6 mg per dose. The age at which it is used varies between products, with some suggesting use beginning at 2, 3, or 4 years of age. Directions vary between gummies, liquid, and tablet forms, but generally suggest administration 30 to 60 min before bedtime. Gummy doses typically range from 0.5 mg to 1 mg, suggested for ages two and older, with 1 to 2 gummies per dose. Liquid and tablet melatonin dosing varies with age: for 3- to 5-year-olds the dose is suggested not to exceed 1 mg; for 6- to 12-year-olds, a 2 mg dose is suggested, and those older than 12 can take 3 mg. It is recommended to administer these forms 30 to 60 min before bedtime [34].

There are differing perspectives on melatonin dosing due to limited evidence, specifically the paucity of randomized control trials. Although weak for recommendation as it is based on 1 study only, the AASM practice guideline suggests that clinicians treat children and adolescents with DSPS (and no comorbidities) with strategically timed melatonin versus no treatment. Based on one randomized, placebo-controlled double-blinded study with participants aged 6–12 years with DSPS and no comorbidities, optimal results were observed at a dose of 0.15 mg/kg, taken 1.5–2.0 h prior to habitual bedtime with consistently nightly timing for period of 6 days (overall quality of evidence -moderate, based on AASM guidelines) [35]. For children and adolescents with DSPS and psychiatric comorbidities, AASM suggested strategically timed fast-release melatonin at dosages ranging from 3–5 mg, taken at 18:00–19:00 for 4 weeks may be effective for children/adolescents (overall quality of studies reviewed was low) [35].

In one study, for the treatment of delayed sleep phase disorder, melatonin was administered to children at doses of 2.5 mg to 3 mg, while adolescents required doses of 5 mg to 10 mg. It was suggested to be taken 30 to 60 min before the desired bedtime to help advance the onset of sleep and address circadian rhythm sleep disorders [36].

In the case of behavioral insomnia, sleep-onset association type, research has explored the use of melatonin in randomized, double-blind, placebo-controlled trials. School-age children were given a 5 mg dose of melatonin at 19:00 for four weeks to improve sleep. A similar study focused on 105 children aged 6 to 12 years with ADHD and sleep-onset insomnia, who received 3 mg or 6 mg of melatonin (adjusted by body weight) or a placebo for four weeks. In an open-label study involving 107 children with ASD, aged two to 18 years, who experienced various sleep problems, melatonin was administered 30 to 60 min before bedtime. Doses for children under six years started at 0.75 mg to 1 mg, with potential dose increases every two weeks. Children aged six and older began with a 1.5 mg dose, with instructions for dose escalation if there was no clinical response. These studies showed an overall 60% improvement in overall perception of sleep by parents, but objectively had notable improvement in sleep onset and latency [36]. 

In one study that observed melatonin use in pediatric ICU and non-ICU settings in a tertiary care center over a 4 year time period, 4.7% of participants were under 1 year of age receiving a mean dose of 0.8 mg. Average dosing was 5 mg usually in patients who were above 12 years of age. High doses of melatonin, 20 to 30 mg, were used several times for inflammation and anesthetic purposes [37].

Nevertheless, there are many different studies with varying dosing guidelines. In general, pediatric dosing consistently ranges between 0.5 mg and 5 mg about 30 to 60 min prior to bedtime. Some studies suggest a maximum dose based on age, which should be considered. 

In terms of alternative non pharmacologic treatment, there is evidence to use cognitive behavioral therapy for insomnia in school aged children. A randomized control trial showed that it had positive effects on sleep latency, waking after sleep onset, and efficiency. In this study, this seems to have had a greater benefit with adolescents who have significant delay in sleep, and not as helpful in those with mild delays in sleep [30]. 

### Adverse Effects and Poisoning

In general, melatonin is not commonly reported to have serious adverse effects but caution is recommended with administration in children and adolescents [35]. The commonly reported adverse effects of melatonin are nightmares, vivid dreams, and extreme sedation [38]. In clinical practice, supratherapeutic levels of melatonin have led to side effects including finding it harder to sleep the next day, having good sleep initiation lasting a few hours and then being unable to return to sleep. MT1 and MT2 receptors are regulated differently in vitro. This might explain why there are inconsistent effects with different dosages [39]. 

A meta-analysis that reviewed controlled trials with melatonin (*n* = 10 studies, over 200 subjects mostly adults) used for ≤3 months showed few reports of adverse events (headache, dizziness, nausea, and drowsiness). In an adult study, a randomized, placebo-controlled trial given for 28-days to the healthy adult male at 10 mg melatonin showed no group differences with respect to adverse effects on polysomnographically recorded sleep, subjective sleepiness, laboratory examinations, or other subjectively recorded events [35].

There are few studies on long-term adverse effects in pediatric and adolescent populations. A long-term study with mean follow-up time of 4 years on pediatric patients with DSPS with ADHD who were given melatonin up to 10 mg showed no serious adverse events. This was based on serial interviews with the children’s parents. A follow-up open-label prospective study on participants with neurodevelopmental disabilities and comorbid DSPS who were given controlled-release melatonin with maximum dosage of 15 mg for up to 3.8 years showed no adverse events [35]. There are also concerns on reproductive hormone effects with administration of melatonin in the pediatric group. However, a study conducted in the general Dutch population showed no significant differences detected in tanner stages (questionnaire based study) in children and adolescents given a mean dose of 3 mg administered for about 3 years during pre-puberty compared to non melatonin users [35].

There has been an increase in melatonin ingestions since the COVID-19 pandemic and likely related to increased accessibility from stay-at-home orders and school closures or increased reported prevalence of mental health disorders related to COVID-19. There is also a noted variability of melatonin content with the most variation found in the chewable formulation, which is most commonly used by children [40]. Addressing this variability, a study found there was a large range from −83% to +478% of the labeled content of melatonin. Lot to lot variability existed too, varying as much as 465%. In this case, serotonin was found in 8 of the supplements at levels from 1 to 75 μg. Since serotonin is a prescription medication, it can be an undesirable addition to melatonin use. In fact, serotonin overdose can occur with low levels and cause serious, even fatal, side effects. It can potentially lead to serotonin syndrome, exacerbated by other common medications such as serotonin reuptake inhibitors, triptans, ondansetron and other related medications [41]. This is very concerning since melatonin is unregulated and there are no restrictions on potential additives in the United States. 

A recent study assessed pediatric melatonin ingestions in a 10 year period between 2012 to 2022 reported to the American Association of Poison Control Center’s National Poison Data System (NPDS). It showed an increase by 530% in unintentional ingestions in children under 5 years of age with 5 of them requiring mechanical ventilation and 2 deaths. Although there were 2 deaths, these findings are isolated reports in the context of thousands of ingestions in a 10 year period. Without full medical records, it is difficult to assess if these were truly correlated to melatonin use or other confounding factors. For those children with symptoms, most were related to gastrointestinal, cardiovascular, or central nervous systems. Fortunately, 82.8% of children were asymptomatic [42]. The most common symptoms of ingestion in another retrospective study were drowsiness and GI upset. During this study, referral was based on an ingestion dose of >80 mg or if the child was symptomatic [43].

In a subsequent study, there were seven deaths of children from 2 months to 3 years of age with exogenous melatonin detected in a toxicology analysis, however, full medical records were not available to assess the context of underlying conditions. Concentrations ranged from 3 to 1400 ng/mL postmortem. However, preterm infants can have a half-life of 17–21 h in contrast to 40 min in adults and children greater than 3 years of age can start to develop a half-life closer to that of an adult. Therefore, detection of melatonin postmortem must be interpreted in the context of each age group’s metabolism. In one case, melatonin was found at 460 ng/mL in post mortem blood of a 2 month old, so we must interpret this based on the average consumption of a 2 month old, which is about 150 mL per feed [40].It appears that melatonin is safe and effective at low doses but very high doses in conjunction with altered metabolism are linked to adverse outcomes. 

According to LactMed, the USDA Drugs and Lactation Database, melatonin references about 1 ng/mL as the increase in breast milk following oral melatonin use. At peak circadian rhythm, human breast milk contains about 24 pg/mL of melatonin. In one study, an 18 month old had platelet aggregation abnormalities causing bleeding. The mother was taking exogenous melatonin for sleep. Once she discontinued for 3 months, the baby’s platelet aggregation was normal and had no bleeding episodes. Although it is hard to say this is a direct cause without knowing other confounding factors, it may be a potential adverse effect to note [9].

In terms of development, a child does not exhibit circadian variation until 9–12 weeks of age. This includes pineal gland development, sleep-wake rhythms, and body temperature rhythms. In one study, after an infant was exposed to artificial light when awake or after a feeding and they were administered melatonin, it demonstrated that it can affect the natural temperature control cycle. For example, a 3 year old male took 3 mg of melatonin daily and experienced hypothermia, abdominal pain, and restlessness that was found to be associated with the melatonin use [40].

Melatonin may be involved in early fetal development, which can have direct effects on the placenta, neuronal development, and possibly a role in establishing diurnal rhythms and synchronizing the fetal biological clock. Maternal melatonin also crosses the placenta and can influence fetal development as well. Studies have confirmed the maternal origin of melatonin also occurs in fetal circulation, however maturation and synchronization of the fetal circadian rhythm has not been studied well [26]. Irregularities in maternal and placental melatonin production have also been shown to be related to spontaneous abortion [27].

Since melatonin is metabolized through CYP1A2 and lesser activity through CYP1A1, CYP1B1, and CYP2C19, clinicians should be mindful of drug interactions within these pathways. For example, an antidepressant fluvoxamine and caffeine are some of the most common drugs to interact with melatonin and can increase levels of melatonin in the body [38].

## 3. Discussion and Conclusions

In the past 10 years, the world has experienced an unprecedented event, the COVID-19 pandemic, which displaced pediatric patients from their normal day-to-day schedules. This generation of patients was removed from school, pursued online learning, and lifestyles were changed without warning. Sleep disturbances including insomnia and disrupted sleep schedules were one of the many concerns stemming from this disruptive historical event as periods of productivity were no longer closely regulated in school or by their parents. Our modern lifestyle also with variable exposure to exogenous light has greatly affected our circadian rhythms and in turn our melatonin production. 

The use of exogenous or supplemental melatonin for sleep regulatory effects appears to help, but with increased use, unintentional ingestion events were also reported to increase substantially in unprecedented numbers. With its widespread use in children and the potential known possible toxicity and side effects, more research needs to be done to recommend stricter guidelines and recommendations on dosing and indications for each age group. It is imperative that appropriate warnings are provided given the recent deaths and adverse effects from toxicology ingestion reports with melatonin detected as a possible factor. 

In the past 10 years, research involving exogenous melatonin continues to demonstrate its value in managing circadian rhythm sleep disorders in pediatric populations. Melatonin use is shown to improve sleep onset latency and total sleep time in various populations and children with co-morbidities like neurodevelopmental disorders. After behavioral interventions related to activities that can affect sleep, sleep hygiene education and elimination of ambient light close to bedtime have been utilized, melatonin can be introduced to aid the body’s natural regulation of melatonin. As discussed in this paper, melatonin has potential for use for various areas of medicine such as fertility, inflammatory conditions, and migraine management. It can also induce side effects in 1–10% of users which are relatively benign (dizziness, diarrhea, vomiting, abdominal pain, constipation, arthralgias, back pain, respiratory infections, and urinary tract infection). Breastfeeding mothers should be mindful when taking exogenous melatonin as it can pass through her milk. When used appropriately and safely with appropriate safeguards no different for other pharmaceutical agents, exogenous melatonin may be a helpful tool for the modern-day pediatric caregiver with the support of the clinician to help guide best therapy options.

## Data Availability

No new data were created or analyzed in this study. Data sharing is not applicable to this article.
